# Can Chest Ultrasound Replace Chest X-ray in Thoracic Surgery?

**DOI:** 10.3390/tomography8040175

**Published:** 2022-08-20

**Authors:** Konstantinos Grapatsas, Vasileios Leivaditis, Benjamin Ehle, Anastasia Papaporfyriou

**Affiliations:** 1 Department of Thoracic Surgery, Klinikum Bielefeld, 33647 Bielefeld, Germany; 2 Department of Cardiothoracic and Vascular Surgery, Westpfalz-Klinikum, 67655 Kaiserslautern, Germany; 3 Department of Thoracic Surgery, Medical Center-University of Freiburg, Faculty of Medicine, 79098 Freiburg, Germany; 4 Division of Pulmonology, Department of Internal Medicine II, Medical University of Vienna, 1090 Vienna, Austria

**Keywords:** chest XR, chest ultrasound, postoperative complications

## Abstract

*Background:* There is growing evidence that supports the use of chest ultrasound (CUS) versus conventional chest X-ray (CXR) in order to diagnose postoperative complications. However, data regarding its use after thoracic surgery are scarce and contradictory. The aim of this study was to conduct a systematic review to evaluate the accuracy of CUS after thoracic surgery. *Methods:* An electronic search in MEDLINE (via PubMed), complemented by manual searches in article references, was conducted to identify eligible studies. *Results:* Six studies with a total of 789 patients were included in this meta-analysis. Performing CXR decreased in up to 61.6% of cases, with the main reasons for performing CXR being massive subcutaneous emphysema or complex hydrothorax. Agreement between CUS and routine-based therapeutic options was, in some studies, up to 97%. *Conclusions:* The selectively postoperative use of CUS may reduce the number of routinely performed CXR. However, if CUS findings are inconclusive, further radiological examinations are obligatory.

## 1. Introduction

In various medical fields, chest ultrasound has proved its efficiency in diagnosing chest pathologies. In intensive care medicine and traumatology especially, CUS is established in the guidelines of every society and shows accuracy in the confirmation of various emergency conditions [1,2,3]. CUS provides a “real-time” exam that allows the investigation of the chest cavity in any postoperative clinical situation. Most data concerning the validity of CUS compared to CXR arise from patients after spontaneous pneumothorax (PTX), chest trauma for ultrasound examination in the intensive care station [4,5]. CUS seems to have certain advantages over CXR. CUS is free of radiation and can be independently and repeatedly performed by examiners by the bedside. Its function depends on interpreting artifacts that are produced when the sound waves reflect on surfaces. However, CUS is not so objective as it is dependent on the examiner and how he interprets an image. Moreover, it requires specific training and knowledge of thoracic physiology [2,3].

In the postoperative course after thoracic surgery, CXR is the standard imaging examination. In some thoracic surgical departments, CXR still remains a routine practice, even in uneventful patients. Efforts and suggestions have been made to reduce the number of postoperative CXR. However, such an action would require close clinical monitoring from experienced surgeons [6]. In this case, the performance of CUS, planned or on demand, could theoretically contribute to the reduction in CXR use. As a result, CUS would be of special importance in the postoperative course after noncardiac thoracic surgery. However, in this surgical field, the number of relevant studies is limited and occasionally, with contradictory results [7,8].

In the current study, we conducted a systematic review and meta-analysis of cohort studies that comprehensively evaluate whether CUS could replace CXR as a postoperative imaging examination after thoracic surgery.

## 2. Materials and Methods

This systematic review and meta-analysis was performed according to the Preferred Reporting Items for Systematic Reviews and Meta-Analyses (PRISMA) statement protocol. We analyzed findings of CUS studies after thoracic surgery and their clinical suggestions.

### 2.1. Search Strategy

We searched MEDLINE (via PubMed) to June 2022 to identify studies relevant to this review. The combination of the following keywords was used as search terms: “chest/thoracic ultrasound”, “thoracic/chest sonography”, “lung/thoracic surgery”, and “lung/thoracic resection”. In addition, the reference lists of the articles detected were further searched by hand to identify additional relevant reports. 

The eligibility of the retrieved studies was independently performed by two authors (Konstantinos Grapatsas and Vasileios Leivaditis). The review authors resolved any difference of opinion through discussion or by appeal to a third review author (Benjamin Ehle) when necessary. Finally, an additional manual search was performed by the two investigators on references from the retrieved studies to identify relevant articles.

### 2.2. Inclusion and Exclusion Criteria

Studies were considered eligible if they referred to postoperative CUS after noncardiac thoracic surgical operations. 

Studies were excluded based on any of the following criteria:

(I) the following article types: reviews, letters, laboratory research, and animal experiments;
(II) if the language was not English;
(III) studies including only patients after cardiac surgery;
(IV) studies from the same institution retrospectively examining the same population.

### 2.3. Quality Assessment

The quality of each included study was assessed using the Newcastle–Ottawa Scale (NOS). Based on the quality of selection, comparability, and exposure, a score with a maximum of 9 points was appointed.

## 3. Results

**Figure 1:**
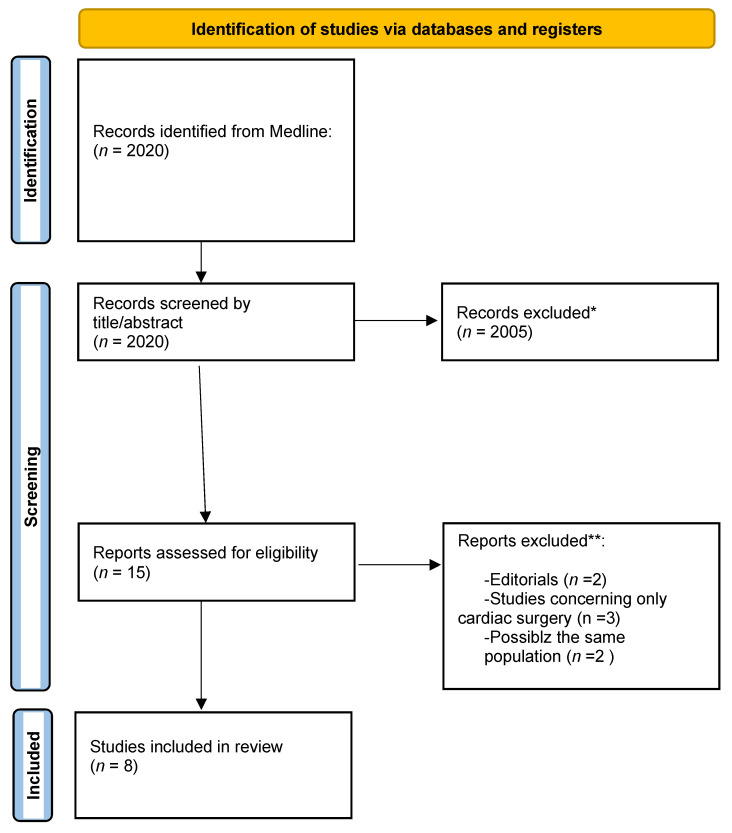
Identification of CUS studies according to Preferred Reporting Items for Systematic Reviews and Meta-Analyses (PRISMA) statement protocol. * If automation tools were used, indicates how many records were excluded by automation tools. ** If automation tools were used, indicates how many records were excluded by human.

After primary retrieval in Medline, a total of 2020 potentially relevant studies were incorporated into our initial study. Then, 2005 articles were excluded as irrelevant by the title or abstract screening. Full texts were retrieved from the remaining 15 studies. Eight of them met all the inclusion criteria in the analysis (Figure 1, Table 1).

We included the data from the study of Touw et al., although they addressed a different category of postoperative patients after cardiothoracic surgery, as the relevant information about CUS in these patients could be used as extrapolation for the current study [9]. However, the studies of Canty et al. [10], Vezzani et al. [11], and Alsaddique et al. [12] also regarding cardiac surgery did not meet the criteria of the study and therefore were excluded.

### 3.1. Characteristics and Qualities of the Included Studies

Studies, that were included, are summarized in Table 1. A total of 730 patients participated in these studies. There were three studies with a size sample of less than 50 patients. All eight studies that were included are cohort prospective studies. Four of them were published after 2018. Major lung resections (lobectomies) varied between studies and ranged between 29–66% of cohort populations [7,8,13,14,15,16,17]. Seven of the eight studies presented were clearly in favor of CUS in the postoperative follow-up of patients after thoracic surgery [8,9,13,14,15,16,17]. Quality assessments of the individual studies are shown in Table 1.

### 3.2. Postoperative Evaluation of PTX

PTX was identified postoperatively in 315 patients with CXR after thoracic surgery. CUS led to the diagnosis of PTX in 161 cases. Most of the cases of PTX had no clinical relevance. The sensitivity and specificity of CUS, especially for PTX, was very high and in some cases reached 100% [14,15]. The agreement between CXR and CUS reached up to 97% in some studies [14].

### 3.3. Postoperative Evaluation of Pleural Effusion (PE)

The diagnosis of postoperative PE was examined in four studies. After thoracic surgical operations, a PE was diagnosed in 368 cases with CXR and in 300 cases with CUS. The diagnostic disagreement between the two studies was minimal and led to no relevant clinical interventions.

### 3.4. Evaluation of Other Chest Pathologies

Other chest pathologies such as postoperative consolidation or hypo-contractility were not thoroughly investigated. Subcutaneous emphysema (SE) was diagnosed more often with CUS. SE was detected in 16 patients with CUS and in 8 with CXR [8].

Postoperative lung consolidation (LC) and lung atelectasis (LA) were only examined in two studies [8,9]. CUS was able to detect more LC than CXR. Twenty patients were detected in both studies with CUS, while all these cases were missed with CXR. CUS also showed a significant advantage in diagnosing LA. In the study of Touw et al., on the day of the operation (Day 0), 154 patients with LA were diagnosed with CUS versus 74 that were diagnosed with CXR [9]. In addition, in the study of Chiappetta et al., a case of a lobar atelectasis in CUS was misinterpreted as hydro-pneumothorax using CXR. In the same study, the complete accordance between the two examinations regarding LA and LC was only 8.33% [8].

Chiappetta et al. investigated the hypo-contractility of the diaphragm after thoracic surgery. CUS detected two additional cases in comparison to CXR. However, the detected cases were without any clinical significance [8].

### 3.5. Detection of Pulmonary Oedema

CUS was more efficient in detecting postoperative pulmonary oedema after thoracic surgery. In the study of Touw et al., CUS showed a trend in diagnosing postoperative pulmonary oedema more often in all three days that an examination was performed (Day 0: 36 cases vs. 26; Day 2: 14 vs. 9; and Day 3: 20 vs. 11) [9]. Galetin et al. detected only one case with pulmonary oedema using CUS that was missed with CXR [14].

### 3.6. Random Findings in Performing CUS

CUS also detected mediastinal shift (*n* = 2) and atrial fibrillation (*n* = 3, one of which was a first-time diagnosis) [8,14].

### 3.7. Overcoming Challenges in Performing CUS

Goudie et al., in the first study that examined the utility of postoperative CUS in thoracic surgical patients, suggested that SE was a relatively common limitation for CUS. The authors detected SE on 110 hemithoraces that restricted visualization with CUS. However, when it was limited to a region of the hemithorax, a partial evaluation was possible [7]. However, Chiappetta et al. reported that the performance of CRX was needed only in four cases with massive SE (25% of all SE cases) [8]. Additional limitations for Goudie et al. were surgical closure sites, dressings, and limited patient mobility as it sometimes made the posterior costodiaphragmatic angle difficult or impossible to visualize [7].

### 3.8. Reduction in CXR by Performing CUS

The performance of CUS resulted in the limitation of postoperative CXR. The use of CUS as imaging for postoperative thoracic surgical patients reduced the necessity for postoperative CXR by up to 86%. More specifically, according to Malik et al., CXR could be decreased by up to 61,6%, while, according to Platella, this reduction could reach up to 86% in some cases [13,17].

## 4. Discussion

To our knowledge, it is the first time that a comprehensive and detailed systematic review and meta-analysis has been performed to evaluate if CUS could replace CRX in the clinical praxis after thoracic surgery. A variety of studies and meta-analysis, such as that of Winker et al., have highlighted the superiority of CUS versus CXR compared to gold-standard CT in detecting lung pathologies in critically ill patients [18]. However, for the postoperative use of CUS in thoracic surgery, only Bhakhir et al. and Nooitgedacht et al. have made editorial suggestions [19,20]. The role of CUS in the postoperative course after lung resection is under discussion. In the existing literature that compares CUS and CXR in internal medicine and traumatology, CUS shows superiority. However, at this point, it should be mentioned that in these patients the CXR was shot in supine position that impaired its sensitivity [21,22].

Although from the study of Goudie et al., there was evidence that CUS could be useful in PTX diagnosis, this trial had some weaknesses because CUS was only performed in the sitting position [7]. Performing CUS in supine position, as in traumatology and intensive care medicine, could have provided better results [21,23]. Specifically, CUS detects lung expansion by evaluating the movement of the pleura against the thoracic wall that occurs with respiration. As a result, “seashore sign” is the granular appearance of the lung in contrast to the motionless portion of the chest wall. In the case of PTX in M-mode, the lack of movement will only display one pattern of parallel horizontal lines above and below the pleural line. This pattern resembles a “barcode” and is often called the “stratosphere sign” [24,25]. The authors suggested that CUS could reduce CXR in patients with a previously ruled out PTX, but they were hesitant in recommending the replacement of CXR by CUS [7]. Five years later, a study from Italy conducted by Chiappetta et al. opposed that idea, suggesting that CXR could be replaced, especially in minor and mini-invasive surgery and in uncomplicated patients, and should be preserved in these cases as a second-level exam [8]. This study evaluated the exhaustiveness of CUS diagnosing PTX in a limited number of patients (*n* = 24) focusing on a selected surgical approach. Although CUS was limited from the presence of subcutaneous emphysema (SE), a CRX was needed in five cases with massive SE (from the 16 cases with SE) [8]. In the same year, the team of Platella supported the results of Chiappetta et al. showing that in only 1% of patients, there was no accordance between CUS and CXR in identifying PTX [13]. On the other hand, Platella et al. addressed the concerns of Goudie et al. that, in some patients, CUS could overestimate PTX, so further radiological examinations before reinsertion of a chest drain for postoperative PTX could be needed [13]. Specifically, Goodie et al. used the absence of lung sliding as a sign for PTX, and no further investigation was conducted in searching for the lung point, which they suggested as a limitation in their study, that could lead to false positive results [7]. Whereas Patella et al. declared that the false positive results in their study could arise from the procedures performed in which a residual empty pleural space, either apical or anterior, is not evident on the CXR and could be considered as normal [13]. As a key process to increase the specificity of CUS in diagnosing PTX, Malik et al. proposed creating a stratified algorithm, in which the center part is the addition of more ultrasound signs that exclude PTX such as lung sliding, B-lines, lung pulse or a more complex algorithm such as the BLUE (Bedside Lung Ultrasound in Emergency) protocol [17]. The BLUE protocol is incorporated within the control of acute respiratory failure and describes the use of ultrasound in critically ill patients in order to diagnose pneumonia, pulmonary edema, COPD exacerbation, pulmonary embolism, or PTX [24,25]. This BLUE protocol was applied in 123 patients by Galetin et al. to diagnose between PTX or other pathologies after anatomical and nonanatomical lung resection. CUS showed a high sensitivity and specificity (100% and 82%, respectively) for large PTX (PTX ≧ 3 cm) and resulted in diagnosing all clinically relevant PTX in this trial [14]. In a newer study from the same institution, postoperative CUS replaced CXR with the same sensitivity for PTX and resulted in equally safe patient management [15]. In their third study, Galetin et al. compared CUS and CRX for the detection of PTX after lung surgery by combining the populations (340 examinations were performed in 208 patients) of two previous prospective trials. The most important outcome of this study was that sensitivity or specificity of CUS could not be influenced by perioperative factors such as age, gender, body mass index, smoking status, or the severity of chronic obstructive pulmonary disease (COPD). In addition, surgical or oncological factors such as previous lung operations, radiation or thoracotomy as surgical approaches showed no significant effect on the specificity and sensitivity of CUS [26].

With regards to chest drain removal, most of the studies agreed that CUS is an efficient and reliable method. Patella et al., in their observational study, investigated the utility of CUS in 50 patients regarding postoperative chest drain removal [13]. The authors suggested the removal of the chest drain if a minimal air leak existed, and CUS confirmed the expansion of the lung. In this way, CRX could be saved. However, in this study, CUS was only performed in a selected population of patients and that could be a bias, as complicated patients such as those with surgical emphysema, severe COPD, chest wall or diaphragm resection were excluded [13]. Similar to this evidence, Chiappetta et al. assessed the efficacy of CUS in removing the chest drain after thoracic surgery but they also focused on the type of surgery. It was suggested that using a localized apical PTX with no air leak, the chest drain could be safely removed [8]. As a result of the above studies, it could be proposed that noncomplicated surgical patients or minimal-invasive operations are the ideal patient groups for postoperative follow-up with CUS [8,13].

It is known that CUS can easy detect a PE even when the amount of fluid is still very low [27]. However, it can be a tricky diagnosis with CXR for patients in an acute setting after surgery, who stay in a lying position. Dzian et al. showed that CUS can safely diagnose PE from the first examination with high sensitivity and specificity (up to 86.2% and 88.4%, respectively) and a good agreement with CXR results [16]. Malik et al. showed that the rates of sensitivity and specificity of CUS for PE were similar to those for PTX (60.9% and 91.3%; and 59.4 and 94.8, respectively) and that the specificity of CUS increased after the first examination [17]. Both studies concluded that a postoperative CUS performed by a thoracic surgeon could safely replace CXR as the primary examination in the postoperative follow-up [16,17].

In 2018, a trial by Touw et al. found a superiority of CUS compared with CXR in detecting PTX or other lung pathologies and for patients undergoing cardiothoracic surgery [9]. By using the BLUE protocol in 177 patients, this study detected earlier and more clinically relevant pulmonary complications with CUS than with CRX. Moreover, regarding the diagnosis of postoperative LA and LC, CUS showed an advantage. In these cases, CUS gave the ability to distinguish if the reason for LA was hypoventilation or lung contusion due to parenchymal resection [9]. The main limitation of this study was that the authors did not compare the results of CUS and CRX with the gold standard method, thorax CT. Furthermore, the subjectivity of the treating physician may be an issue, though the performance of CUS by multiple investigators, with different levels of experience, thus mirroring daily clinical practice, may overpower it [9,28,29]. More double-blind, randomized trials with postoperative patients after cardiothoracic surgery are needed so that we can come to safer conclusions, but regarding the above studies, we can recommend CUS as the primary imaging technique to detect chest pathologies and help in bedside decision making.

### 4.1. Limitations of This Meta-Analysis

The most important limitation of this meta-analysis is the heterogeneity of the study populations. In some studies, major lung resections were the majority of the operations, whereas in other cases, they were limited [8,13]. The differences in the extent of the operations as well as the different surgical approach (open vs. VATS) could result in different ultrasound findings. In addition, in some studies, CUS was absolutely performed in selected patients [13,14]. As a result, the conclusions concerning CUS in unselected patients with, for example, lung emphysema could not be made. Furthermore, the performance of CUS and the exclusion of the chest pathologies were made with different algorithms. In all included studies, the timepoint or the frequency of examination, the definition of chest pathologies or the position of the patient during the examination were not similar. For example, Goudie et al. performed CUS several times after surgery [7]. In the study of Patella et al., CUS was performed after the removal of the chest tube [13]. In some patients, CUS was performed within 48 h after surgery [8]. Similarly, the position of patients differed. In the study of Goudie et al., CUS was performed in sitting or at least 45o semi-supine position [7]. Patella et al. performed CUS also in sitting position [13]. This heterogeneity concerning the performance of CUS could have ended in different results. For example, in the above-mentioned studies, the sensitivity of CUS for PTX significantly differed (0.2 vs. 1.00). In addition, the lack of a standardized algorithm in all studies or a widely accepted protocol could have resulted in different findings. Malik et al. proposed an algorithm in order to have a standardized diagnostic procedure [17]. Patella et al., in order to better evaluate the significance of a postoperative PTX, performed CUS using two different anatomical evaluation points (2nd and 3rd intercostal space) [13]. Galetin et al. used, as a sign, the lung pulse for ruling out PTX [14,15]. Moreover, the use of thorax CT as the gold standard method to assess the sensitivity and specificity of CUS compared to CXR has not been applied in all studies [8,9,13]. Thus, the results could be controversial.

The urge for new trials with better methodology and larger populations is undeniable. However, from the present studies, independent of the CUS protocol/algorithm, imaging with CXR could be replaced or reduced by performing a CUS. Consequently, it could be suggested that the use of CUS postoperatively as a primary tool for the diagnosis of lung pathologies has many advantages for patients after lung resection. CUS does not expose patients to radiation. It can be performed at bedside, and it is repeatable. The fact that CUS is performed at bedside is also comfortable for the patient. Furthermore, a high number of CUS could be performed at low cost and without overdosing the health care system. Finally, if the CUS-examination is performed by a thoracic surgeon that already has the clinical information concerning the postoperation anatomical changes, then the need for additional imaging examinations could be further reduced.

### 4.2. Future Directions

Recently, noninvasive, low-cost and free of radiation evaluation methods such as CUS have gained more access in daily clinical practice. More and more specialists have been trained to recognize pathologies in their field of interest, although educational programs officially accepted by different medical societies (pulmonologists, thoracic surgeons, etc.) should be established. Furthermore, artificial intelligence with the use of complex algorithms could provide support in analyzing all the existing data from a patient along with ultrasound imaging and make the most accurate diagnosis. As it is important to avoid subjectivity and interpret images more objectively, the insertion of an artificial intelligence program can help in accomplishing this, while, in parallel, the repeatability of a certain result could be validated. New controlled, randomized trials with postoperative patients should check this possibility in order to optimize this diagnostic tool in the hands of clinicians.

## 5. Conclusions

CUS could replace CRX in clinical praxis after thoracic surgery, even after major lung resections. Further studies in the field could focus on strengthening the position of CUS in decision making rather than directly comparing CUS with CXR. The use of standardized ultrasound protocols, such as the BLUE protocol, could reduce any indeterminate results as well as conducting unnecessary CXR. However, it should be the physician, with the knowledge of the limitations and advantages of each method, who should choose which examination could benefit the patient in each case.

## Figures and Tables

**Table 1:** Basic characteristics of the included studies.

Study	Patient Source	Total Number of Patients	Number of Lobectomies	Identified PTX with CXR vs. CUS	Identified PE with CXR vs. CUS	Key Results	NOS-Score
Goudie, 2011	Canada	120	36	157 vs. 29	148 vs. 118	-PE sensitivity: 83%, specificity: 59% -PTX: sensitivity: 21%, specificity: 95% -adequate method to evaluate PE, uncertain for PTX -postop. CUS may reduce CRX if previously PTX is ruled out -CUS has not have high enough accuracy to replace CXRs. -Limitation: -CUS only in sitting position -lung point not always searched	7
Patella, 2017	Switzerland	50	33	15 vs. 24		-CUS for PTX: -71% positive predictive value -100% negative predictive value -86% CRX saved	7
Chiapetta, 2018	Italy	24	6	0 vs. 11	0 vs. 5	-CUS exhaustive in -67% cases of open surgery -85% cases of VATS -CXR needed only in 20.8% due to massive subcutaneous emphysema	8
Malik, 2020	Slovakia	297	45	69 vs. 51	169 vs. 117	-CUS sensitivity and specificiity for -for PTX up to 59.4% and 94.8% -for PE up to 60.9% and 91.3% -61.6% CXR saved -Non-physiologic finding -> other imaging modality	7
Dzian, 2021	Slovakia	48				-CUS sensitivity for -PTX up to 58.5% -PE up to 86.2% -2 PTX missed from CRX, all other mismatch clinical irrelevant -CUS could reduce CRX -BLUE protocol	6
Galetin, 2019	Germany	123	44	44 vs. 26		-CUS sensitivity and specificity for large PTX 100% and 82% -No clinically relevant PTX missed. -Agreement between CUS and routine-based therapeutic decisions ≧ 97%	8
Galetin, 2021	Germany	68	31	23 vs. 18		-CUS sensitivity and specificity for PTX 81% and 81–100%	8
Touw, 2019	Netherlands	177	0	7 vs. 2	51 vs. 60	-CUS detected more clinically-relevant postoperative pulmonary complications and earlier than CXR -BLUE protocol	8

PE: pleural effusion; PTX: pneumothorax; CUS: chest ultrasound; VATS: video-assisted thoracic surgery.

## Data Availability

The data presented in this study are available upon request from the corresponding author.
